# Pediatric Firearm Injury-Related Emergency Department Visits in Florida: A Regional Analysis by Age Group and Insurance Status

**DOI:** 10.7759/cureus.87425

**Published:** 2025-07-07

**Authors:** Claudia A Serna, Oscar Arevalo

**Affiliations:** 1 Public Health, Nova Southeastern University, Davie, USA; 2 Pediatric Dentistry, Nicklaus Children's Hospital, Doral, USA

**Keywords:** emergency department, firearm, geographic regions, pediatric, sociodemographic factors

## Abstract

Objective: This study aimed to analyze pediatric firearm-related emergency department visits in Florida. We stratified the data by region, age group, and insurance status to identify demographic and geographic disparities and inform targeted public health interventions.

Methods: Data were obtained from the Florida Agency for Health Care Administration for the years 2013 to 2018, focusing on ambulatory emergency department (ED) visits involving patients aged 19 years or younger with a principal diagnosis of firearm-related injury. These cases were identified using the International Classification of Diseases, Ninth Revision, Clinical Modification, and the International Classification of Diseases, Tenth Revision, Clinical Modification, diagnostic codes. Florida was divided into 11 geographic regions based on the patient’s county of residence. Pediatric firearm injury visit rates were calculated per 10,000 population. The total number of ED visits for firearm-related injuries among pediatric patients from 2013 through 2018 was summed and divided by regional population estimates obtained from the Florida Community Health Assessment Resource Tool Set for the corresponding years. To assess statistically significant differences across regions and age groups, we employed the chi-square (χ²) test, considering a p value of ≤0.05 as statistically significant. Additionally, we examined the percentage distribution of pediatric firearm injuries by insurance status, categorized as Medicaid, self-pay, private insurance, other, and uninsured.

Results: The highest rates of firearm-related injuries were observed among older children aged 15-19 years and among uninsured patients. Statistically significant differences in injury rates were identified across all geographic regions when stratified by age groups.

Conclusions: This study reinforces existing evidence that older children and uninsured older children are disproportionately affected by firearm injuries. These findings highlight the need for targeted public health interventions and further research focused on high-risk pediatric populations.

## Introduction

Pediatric firearm-related injuries are a major cause of morbidity and mortality in the United States and are thus a public health concern [1-17]. In 2019, approximately 115,000 firearm-related injuries were reported in the United States [1].

For every child killed due to a firearm-related incident, a significantly greater number are severely injured. Approximately half of those hospitalized with a firearm-related injury are discharged with increased health risk behaviors, poor health status, lifelong physical and mental consequences, and disabilities [1-5,8-11]. These disabilities include limitations in vision, hearing, speech, bladder, and bowel control as well as in daily living activities [9].

Research conducted in the United States indicates that socioeconomic disparities significantly mediate the impact of gun violence, with adolescents (15-17 age group) experiencing a disproportionate burden [1,3-14,18]. That is why the purpose of this study is to identify differences in pediatric firearm emergency department (ED) visits across Florida regions, considering age groups and insurance status.

## Materials and methods

This research project received approval from the Institutional Review Board at Nicklaus Children's Hospital. The data analyzed in this study were sourced from ambulatory ED discharge records maintained by the Florida Agency for Health Care Administration (AHCA) [19]. These records include all ED visits with registrations occurring between 2013 and 2018 and contain patient demographic information, diagnostic codes for firearm-related injuries based on the International Classification of Diseases, Ninth Revision, Clinical Modification (ICD-9-CM) and International Classification of Diseases, Tenth Revision, Clinical Modification (ICD-10-CM), as well as information on the patients’ primary insurance payers [20]. The study included patients aged 0-19 years who presented to the ED with a documented firearm-related injury diagnosis during the study period. Only records with complete demographic and insurance information were considered. To ensure consistency, only ambulatory ED discharges were included in the analysis. Patients were excluded if they were over 19 years of age, if their ED visit did not involve a firearm-related injury, or if their records lacked essential demographic or insurance data. Inpatient admissions and transfers that were not classified as ambulatory ED discharges were also excluded from the study.

To ensure an adequate sample size for the primary analysis, we merged data from six calendar years: 2013, 2014, 2015, 2016, 2017, and 2018. To identify regions with higher prevalence and rates of pediatric firearm injuries, we divided the state of Florida into 11 service areas as designated by the Agency for Health Care Administration (AHCA) [21]. AHCA is responsible for administering the Florida Medicaid program, licensing and regulating health care facilities, and providing the public with information about the quality of care available in the state. Pediatric firearm injury visits were identified based on the primary diagnosis assigned by ED physicians, using the ICD-9-CM and ICD-10-CM codes. The AHCA used ICD-9-CM for the years 2013 and 2014, as well as the first three quarters of 2015, before transitioning to ICD-10-CM for the remainder of the study period.

For the purposes of this analysis, a firearm injury visit was operationally defined as one in which the admitting diagnosis included any of the following ICD codes: E92.21/W33.01XA, E92.22/W33.02XA, E92.23/W33.03XA, E92.28/W33.09XA, E92.29/W33.10XA, E95.50/X72.XXXA, E95.51/X73.0XXA, E95.52/X73.1XXA, E95.53/X73.2XXA, E95.54/X73.8XXA, E96.50/X93.XXXA, E95.59/X74.9XXA, E96.51/X94.0XXA, E96.52/X94.1XXA, E96.53/X94.2XXA, E96.54/X94.8XXA, E97.0/Y35.001A, E97.94/Y38.4X1A, and E98.50/Y22.XXXA [22].

Since population size varies across geographic regions and over time, firearm injury rates provide a more accurate basis for comparison. In this study, firearm injury rates were calculated as the number of injuries per 10,000 individuals. These rates were further stratified by age groups: 0-4, 5-9, 10-14, and 15-19 years. To assess statistically significant differences across regions and age groups, we employed the chi-square (χ²) test. A p value of ≤0.05 was considered statistically significant. Additionally, we analyzed the percentage distribution of pediatric firearm injuries by insurance status, categorized as Medicaid, self-pay, private insurance, other, and uninsured [20].

## Results

After merging the calendar years 2013 through 2018, in terms of age groups, the rate of ED pediatric firearm- related injury visits per 10,000 population was 0.04 for zero to four years old, 0.04 for five to nine years old, 0.20 for 10-14 years old, and 1.60 for 15-19 years old. For children aged zero to four years in Region 3, which includes Alachua, Bradford, Citrus, Columbia, Dixie, Gilchrist, Hamilton, Hernando, Lafayette, Lake, Levy, Marion, Putnam, Sumter, Suwannee, and Union, the rate is 0.09. For ages five to nine years in Region 1, comprising Escambia, Okaloosa, Santa Rosa, and Walton, the rate is 0.13. For ages 10-14 years in Region 2, which includes Bay, Calhoun, Franklin, Gadsden, Gulf, Holmes, Jackson, Jefferson, Leon, Liberty, Madison, Taylor, Wakulla, and Washington, the rate is 0.34. Lastly, for ages 15-19 years in Region 4, covering Baker, Clay, Duval, Flagler, Nassau, St. Johns, and Volusia, the rate is 2.44 [21]. After performing a χ² test for age groups, statistically significant differences were observed in the prevalence of firearm-related injuries among all age groups across all regions (Table 1).

**Table 1:** Firearm injuries among Florida youth: ED visit rates by region and age (2013-2018) Statistical significance across regions and age groups was evaluated using the chi-square (χ²) test ^*^Statistical significance at p < 0.001 ED: emergency department

Florida region		Frequency 0-4 pediatric ED firearm-related injuries	Rates 0-4 pediatric ED firearm-related injuries	Frequency 5-9 pediatric ED firearm-related injuries	Rates 5-9 pediatric ED firearm-related injuries	Frequency 10-14 pediatric ED firearm-related injuries	Rates 10-14 pediatric ED firearm-related injuries	Frequency15-19 pediatric ED firearm-related injuries	Rates 15-19 pediatric ED firearm-related injuries	p value
Region number	Counties									
1	Escambia, Okaloosa, Santa Rosa, and Walton	1	0.01	7	0.13^*^	15	0.29	71	1.26	<0.001
2	Bay, Calhoun, Franklin, Gadsden, Gulf, Holmes, Jackson, Jefferson, Leon, Liberty, Madison, Taylor, Wakulla, and Washington	2	0.04	3	0.06	17	0.34^*^	82	1.36	<0.001
3	Alachua, Bradford, Citrus, Columbia, Dixie, Gilchrist, Hamilton, Hernando, Lafayette, Lake, Levy, Marion, Putnam, Sumter, Suwannee, and Union	9	0.09^*^	6	0.05	18	0.17	102	0.9	<0.001
4	Baker, Clay, Duval, Flagler, Nassau, St. Johns, and Volusia	6	0.04	7	0.04	47	0.32	360	2.44^*^	<0.001
5	Pasco and Pinellas	2	0.02	1	0.01	4	0.04	131	1.48	<0.001
6	Hardee, Highlands, Hillsborough, Manatee, and Polk	6	0.03	6	0.03	38	0.2	350	1.89	<0.001
7	Brevard, Orange, Osceola, and Seminole	5	0.02	10	0.05	27	0.13	255	1.24	<0.001
8	Charlotte, Collier, DeSoto, Glades, Hendry, Lee, and Sarasota	6	0.06	3	0.03	11	0.11	151	1.51	<0.001
9	Indian River, Martin, Okeechobee, Palm Beach, and St. Lucie	9	0.07	6	0.04	26	0.19	229	1.68	<0.001
10	Broward	2	0.01	2	0.01	12	0.09	145	1.08	<0.001
11	Miami-Dade and Monroe	7	0.03	7	0.03	61	0.32	393	2.03	<0.001
Total		55	0.04	58	0.04	276	0.20	2,269	1.60	<0.001

Regarding insurance status, uninsured individuals had the largest percentage distribution in Region 10 (Broward), with 30.4%. This was followed by Others in Region 4 (Baker, Clay, Duval, Flagler, Nassau, St. Johns, and Volusia) with 21.2%, Medicaid in Region 11 (Miami-Dade and Monroe) with 19.19%, and Private in Region 6 (Hardee, Highlands, Hillsborough, Manatee and Polk) with 18.5 (Table 2) [21].

**Table 2:** Pediatric firearm injuries in Florida: emergency department visits by region and insurance status (2013-2018) Percentage distributions were evaluated by insurance status: Medicaid, self-pay, private insurance, other, and uninsured ^*^Values marked with an asterisk indicate a significantly higher percentage

Florida region	Medicaid, n (%)	Self-pay, n (%)	Private, n (%)	Others, n (%)	Uninsured, n (%)	
						1	62 (3.7)	14 (2.8)	12 (3.5)	6 (5.1)	0 (0)	
2	56 (3.3)	22 (4.4)	22 (6.4)	3 (2.5)	1 (4.3)	
3	72 (4.3)	37 (7.5)	21 (6.1)	5 (4.2)	0 (0)	
4	256 (15.3)	74 (14.9)	62 (17.9)	25 (21.2)^*^	3 (13)	
5	91 (5.4)	26 (5.3)	16 (4.6)	4 (3.4)	1 (4.3)	
6	239 (14.3)	71 (14.3)	64 (18.5)^*^	23 (19.5)	3 (13)	
7	184 (11)	56 (11.3)	46 (13.3)	10 (8.5)	1 (4.3)	
8	121 (7.2)	27 (5.5)	13 (3.8)	7 (5.9)	3 (13)	
9	173 (10.3)	44 (8.9)	40 (11.6)	12 (10.2)	1 (4.3)	
10	89 (5.3)	45 (9.1)	16 (4.6)	4 (3.4)	7 (30.4)^*^	
11	333 (19.9)^*^	79 (16)^*^	34 (9.8)	19 (16.1)	3 (13)	
Total	1,676 (63)	495 (18)	346 (13)	118 (4)	23 (2)	

## Discussion

Nonfatal firearm injury data sources come from hospital-level data from EDs or trauma centers [5]. These nonfatal firearm injuries reported among American children can be preventable [2]. In our study, the rate of firearm-related injuries did not follow a uniform pattern across the state, as there were substantial variations among Florida regions. The highest rates of firearm-related injuries among children 15-19 years of age were in Region 4 (Baker, Clay, Duval, Flagler, Nassau, St. Johns, and Volusia) [21]. Duval County (Jacksonville area) had the highest gun homicide rate in the state in 2022, at twice the state average [23].

In addition, the cost of medical care for the pediatric population injured by firearms is determined by several financial sources, including Medicaid, private, and out-of-pocket expenses [2]. This study found that most children injured by firearms in Florida were uninsured in Region 10, which is Broward County. These results are consistent with those of previous studies that demonstrated that firearm-related injuries among the pediatric population possess a substantial financial burden on the United States healthcare system among children 15-19 years of age and those who live in large metropolitan areas in the United States [1-6,9-13,15-18,24,25].

The findings of this study should be interpreted in light of several limitations. First, the data were drawn from specific geographic regions and are not nationally representative, which limits the generalizability of the results to other areas of the United States. Second, the analysis relied solely on the patient’s reported reason for the visit (admitting diagnosis) and the clinician’s principal diagnosis code, which may not fully reflect the complexity of each case. Third, key sociodemographic variables were not included in the analysis, despite their potential influence on ED utilization. Fourth, each ED visit was treated as an independent event, and the dataset did not allow for linking multiple visits by the same individual. Consequently, it was not possible to distinguish between initial and repeat visits, limiting the ability to assess patterns of recurrent ED use.

## Conclusions

To our knowledge, this is the first study to examine differences among children in Florida regarding firearm-related injuries presenting to the ED. Our findings reveal that older adolescents, particularly those aged 15-19, and uninsured individuals are more likely to visit the ED for such injuries. Additionally, this study offers a unique perspective that enhances the understanding of firearm injuries in regions with high incidence rates. It also provides valuable insights to inform targeted prevention strategies in schools and communities, as well as local policy responses, such as safe firearm storage laws.

Firearm violence is preventable, so understanding the nature and impact of the problem is the first step toward establishing preventive initiatives. Future studies should explore characteristics at the county and city levels to design targeted multidimensional interventions focused on systemic changes in the individual, family, community, and policy to reduce the burden of gun violence among older children.
